# Drought priming mechanisms in wheat elucidated by *in-situ* determination of dynamic stomatal behavior

**DOI:** 10.3389/fpls.2023.1138494

**Published:** 2023-02-17

**Authors:** Mengxiang Yang, Jiawei He, Zhuangzhuang Sun, Qing Li, Jian Cai, Qin Zhou, Bernd Wollenweber, Dong Jiang, Xiao Wang

**Affiliations:** ^1^ Key Laboratory of Crop Ecophysiology, Ministry of Agriculture, National Technique Innovation Center for Regional Wheat Production, Nanjing Agricultural University, Nanjing, China; ^2^ Department of Agroecology, Aarhus University, Slagelse, Denmark

**Keywords:** wheat, drought priming, tolerance, recovery, stomatal

## Abstract

Stomata play a critical role in balancing photosynthesis and transpiration, which are essential processes for plant growth, especially in response to abiotic stress. Drought priming has been shown to improve drought tolerance. Lots of studies have been done with the response of stomatal behavior to drought stress. However, how the stomatal dynamic movement in intact wheat plants response to drought priming process is not known. Here, a portable microscope was used to take microphotographs in order to *in-stiu* determination of stomatal behavior. Non-invasive micro-test technology was used for measurements of guard cell K^+^, H^+^ and Ca^2+^ fluxes. Surprisingly, the results found that primed plants close stomatal much faster under drought stress, and reopening the stomatal much quicker under recovery, in relation to non-primed plants. Compared with non-primed plants, primed plants showed higher accumulation of ABA and Ca^2+^ influx rate in guard cells under drought stress. Furthermore, genes encoding anion channels were higher expressed and K^+^ outward channels activated, leading to enhanced K^+^ efflux, resulting in faster stomatal closure in primed plants than non-primed plants. During recovery, both guard cell ABA and Ca^2+^ influx of primed plants were found to be significantly reducing K^+^ efflux and accelerating stomatal reopening. Collectively, a portable non-invasive stomatal observation of wheat found that priming promoted faster stomatal closure under drought stress and faster reopening during post-drought recovery in relation to non-primed plants, thereby enhancing overall drought tolerance.

## 1 Introduction

Wheat is one of most important cereal crops worldwide, and plays a dominant role in maintaining food security with the increasing world population. Following the global climate change, the frequency and severity of the extreme stress events, are predicated to be greatly increased. The changing climate obviously raises the risk of cereal supply and global food security. Drought is one of the major limiting factors for wheat production worldwide (Semenov et al., 2014). Global climate models predicting increased frequency, duration and severity of drought episodes have been confirmed in practice, increasing the risk of yield losses under the future climatic conditions (Senapati et al., 2019). Therefore, it is required to enhance wheat stress tolerance in order to sustain crop yield and food security worldwide under the future climate.

Drought priming has been shown to be a potential strategy for improving crop production under drought by enhancing photosynthesis, antioxidant capacity and osmotic adjustments in wheat (Wang et al., 2014a). The central function of stomata is to regulate CO_2_ uptake and H_2_O loss (Wall et al., 2022). The degree of opening of stomata is essential for balancing plant photosynthesis and transpiration, thereby mediating plant adaptation to abiotic stress episodes (Xie et al., 2022). Under drought stress, plants have to make a trade off – either open stomata for optimal photosynthesis, or close them to save water (Kollist et al., 2014). Faster responsiveness of stomata to stress events could improve intrinsic water use efficiency (Drake et al., 2013; Wang et al., 2014b; Lawson and Vialet-Chabrand, 2019). It has been realized that stress tolerance should not only be evaluated by the response during a drought episode but should also include the plant response during post- drought recovery (Visentin et al., 2020; Vilonen et al., 2022). The fine-tuning of this process probably differs between drought stress and recovery and depends on the plant species, the growth stage and the severity of drought stress during stress and recovery periods. However, until now, the results were mainly get by isolate the stomatal through the epidermal strip cells in model plants, while the stomatal dynamic movement in intact plant are not observed.

Stomatal movement is a guard cell-controlled process, guard cells sense changes under drought stress is mediated by hormone, secondary messengers, and ion channel regulation (Roelfsema and Hedrich, 2005; Lawson and Matthews, 2020). The abscisic acid (ABA) is well known plant hormone in regulating stomatal movement under drought stress. ABA-induced stomatal closure is mediated by ion efflux from guard cells. Stomatal opening is driven by the accumulation of K^+^ and sugars in guard cells, the activation of anion channels plays a lead role in stomatal closure. The activity of plant inward-rectifying K^+^ channels is regulated by the calcineurin B-like (CBL) protein–CBL-interacting protein kinase (CIPK) module (CBL–CIPK), changing cytosolic Ca^2+^ concentrations caused by drought (Luan, 2009; Pantoja, 2021). In *Arabidopsis*, Ca^2+^ can contribute to amplifying ABA responses *via* ABA enhancement in the Ca^2+^ sensitivity of S-type anion activation and K^+^ inward channel deactivation in guard cells (Kim et al., 2010; Brandt et al., 2015; Huang et al., 2019). However, little is known how drought priming influences stomatal movement under the reoccurred drought stress and the post-drought recovery in wheat guard cells.

The objective of this study is to *in situ* determine the stomatal dynamic behavior and the underlying physiological mechanisms during a ‘drought-priming–recovery-drought-stress-recovery’ process in wheat. Therefore, in this study, the portable microscope was used to take microphotographs, and dynamic stomatal aperture rate was analyzed the dynamics of stomatal behavior during Furthermore, the content of ABA, ion flux and the expression of related ion channels in guard cell were analyzed to investigate the physiological mechanisms of dynamic stomatal behavior during a ‘drought-priming–recovery-drought-stress-recovery’ process.

## 2 Material and methods

### 2.1 Experimental design

Uniform seeds of winter wheat (*Triticum aestivum* L. cv. Yangmai 16) were surface sterilized with 10% H_2_O_2_ solution for 10 min and washed several times with sterile distilled water. Uniform germinated seeds were selected and grown on a germination net at 20°C. After 14 days, uniformed selected seedlings were cultivated in boxes (40 cm × 35 cm × 18 cm) containing Hoagland solutions, under a controlled environment (day/night temperature at 20°C/18°C), and 14 h photoperiod with photosynthetically active radiation at 350 μmol m^−2^ s^−1^. The Hoagland nutrient solution was renewed every three days and aerated over the completely experimental period. In total, 60 boxes with 30 plants each were used.

At the three-leaf stage, drought priming (P) was applied with 10% PEG-6000 (water potential at −0.58 MPa) for 24 h, after which all plants were ‘recovered’ with normal Hoagland solution for eight days. Then, half of the plants were exposed for a drought stress treatment with 20% PEG-6000 (water potential at −0.96 MPa) for 24 h, and the other half was used as control. Therefore, three treatments were formed after drought stress: CK, non-priming and non-stressed; CD, non-priming plus drought stress; PD, priming plus drought stress.

14 specific time points were picked for the following physiological analyses: 0 h, 1 h, 5 h, 24 h after drought priming; 5 h and 24 h after recovery; 0 min, 5 min, 15 min, 30 min, 60 min after drought stress; and 5 h, 24 h, 48 h after recovery from drought stress.

### 2.2 Water potential and leaf photosynthesis

After the priming and stress treatments, the last fully expanded leave were used to measure leaf water potential and photosynthesis according to Wang et al., 2021. Water potential was measured with dew point microvolt-meter (Wescor Inc., Logan, USA) and photosynthesis, transpiration and stomatal conductance with a portable photosynthesis system (LI-6800, LI-COR Biosciences, Lincoln, USA). All the measurements were taken from 9:00-11:30 a.m., with the CO_2_ concentration set at 400 µmol mol^-1^.

### 2.3 Dynamic monitoring of stomatal movement

We have developed an *in situ* stomatal observation system to investigate the stomatal apertures using portable microscopes in intact wheat leaves (Sun et al., 2021), enabling the recording of the stomatal movement in intact plants under drought stress. Drought priming and drought stress started at 9:00 am on the first day and stopped at 9:00 am on the second day (start of the recovery stage). The last fully expanded leaves were used to take microphotographs with the portable microscope Anyty X600 WM601WIFI (3R Eddytek Corp., China). The magnification was 400-600 times (adjusted according to the actual situation), and the stomatal opening rate (SOR) was analyzed according to the method of (Sun et al., 2019). Three biological replicates were performed. The resulting photos were analyzed by the Image-Pro Plus 6.0 software (Media Cybernetics, Silver Springs, MD, USA) to measure the stomatal aperture length and width, and the resulting stomatal aperture was calculated as aperture width/aperture length (Allen et al., 2000).

### 2.4 Detection and quantification of ABA in guard cells

The ABA content of the leaf epidermis (0.1 g) was determined with ABA ELISA Detection Kits according to the user’s manual (Nanjing Jiancheng Bioengineering Institute, China). ABA quantification was determined using the Multimode Plate Reader Label-free System (PerkinElmer, Wellesley, MA, USA).

### 2.5 *In situ* measurements of guard cell K^+^, H^+^ and Ca^2+^ fluxes by non-invasive micro-test technology

In guard cells, K^+^, H^+^ and Ca^2+^ fluxes were measured using non-invasive micro-test technology (Beijing, China). A segment of leaf epidermis (10 mm × 4 mm) was immersed in measuring solution (0.1mM KCl, 0.1mM CaCl_2_, 0.1 mM MgCl_2_, 0.5 mM NaCl, 0.3 mM MES, 0.2 mM Na_2_ SO _4_, pH 6.0) and fixed horizontally on the bottom of the petri dish using transparent rubber crossbars. K^+^-, H^+^-, Ca^2+^- microsensors were used to determine the ion fluxes. The preparations and calibrations of the ion microsensors was according to Sun et al. (Sun et al., 2009). The ion microsensors were placed about 3 μm above the surface of the tissue and moved repeatedly between two points (10 μm in distance) in a vertical direction to the surface of the tissue. Based on this procedure, the concentration gradients of the target ions were determined, and the ion flux data was recorded every four seconds, and data within five minutes was collected for each sample in this experiment.

### 2.6 Gene expression

The leaf epidermis tissue (100 mg) was homogenized with liquid nitrogen, and total RNA was extracted using Trizol reagent (Invitrogen, San Diego, CA, USA) according to the manufacturer’s protocol. First-strand cDNA was synthesized following the instructions of the HiScript III RT SuperMix for qPCR (Vazyme). Real-time quantitative PCR was performed using ChamQ SYBR qPCR Master mix (Vazyme) in a Bio-Rad iCycler iQ5 fluorescence real-time PCR system (Bio-Rad, Hercules, CA, USA). The annealing temperatures of the target genes were all optimized to 58°C. The primers for quick-activating anion channel 1 (*QUAC1*), slow-activating anion channel (*SLAC1*), malate transport ATPase (*ABCB14*), potassium ion inflow channel (*KAT1*), potassium ion efflux channel (*KOR1*), calcineurin binding protein (*CBL1*), calmodulin kinase (*CIPK23*) are shown in **Table S1**. To normalize results, the Cq (quantification cycle value) was calculated as the relative expression level of each target gene and the housekeeping gene (*Actin*) in each sample.

### 2.7 Statistical analysis

The data were processed using Microsoft Excel 2010. A One-way ANOVA was conducted to analyze the difference between treatments by Sigmaplot 12.5 Systat Software, and significant differences were identified at the 0.05 probability level (p) by least significant difference test (LSD). The raw picture of the stomatal movement was captured at an interval of six seconds, an interval of one minute data was used to plot, figures was made by using Python 3.7 with Matplotlib and Pandas libraries.

## 3 Results

### 3.1 Stomatal behavior

**Figure 1:**
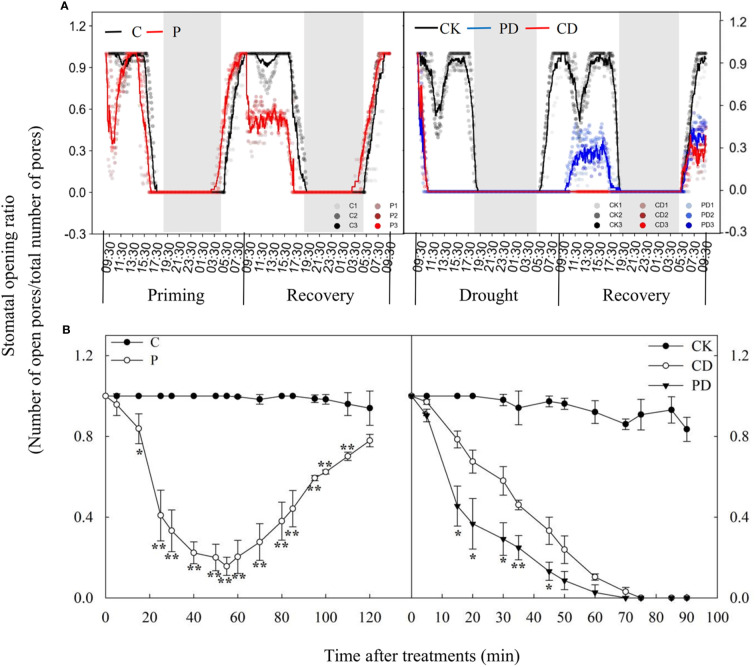
Dynamic responses of stomatal opening ratios during “drought priming-recovery-drought stress-recovery” in wheat. **(A)** Stomatal opening ratio during “drought priming-recovery-drought stress-recovery”. **(B)** Stomatal opening ratios of drought priming within the first 2h and of drought stress within the first 1.5h. The horizontal axis denotes time in **(A)** and treatment time (min) in **(B)**. Drought priming and drought stress start at 9:00 am on the first day and stop at 9:00 am on the second day (start of the recovery stage). Lines are an average of three independent replicates. The white and black rectangles represent day and night, respectively. C, no drought priming; P, priming; CK, no drought priming + no drought stress; PD, priming + drought stress; CD, no priming + drought stress. *p < 0.05; **p < 0.01.

Drought priming and drought stress significantly decreased leaf water potential, stomatal conductance, photosynthesis rate and dry biomass. However, primed plants (PD) could maintain higher leaf water potential, leaf gas exchange and dry biomass (**Table 1**). Drought priming (P) decreased the SOR especially during first 2 hours compared to the control plants (C). The stomata of primed plants (P) closed earlier in the evening and opened earlier in the morning during drought priming, The SOR was significantly lower in P than in C in the first 9 h of recovery (**Figure 1**).

**Table 1:** The leaf water potential, leaf dry biomass, photosynthesis (Pn) and stomatal conductance (Gs) under drought priming and drought stress in wheat." NOTE: C, no drought priming; P, priming; CK, no drought priming + no drought stress; PD, priming + drought stress; CD, no priming + drought stress.

Treatments	Leaf water potential (Mpa)	Leaf dry biomass (g·plant^-1^)	Pn (µmol CO_2_ m^-2^s^-1^)	Gs (mol H_2_O m^-2^s^-1^)
C	-0.43a	0.15a	13.82a	0.46a
P	-0.68b	0.13b	9.6b	0.22b
CK	-0.55a	0.51a	18.29a	0.49a
CD	-1.5c	0.46c	10.6c	0.15c
PD	-0.95b	0.49b	13.73b	0.22b

Drought stress induced closure of the stomata within 5 min and was completed within 60 min, the SOR in drought primed plants (PD) was much lower than non-primed plants (CD). During recovery, stomatal opening in PD happened after 30 min recovery, while no stomatal reopening was found in CD until to the second day (**Figure 1**).

**Figure 2:**
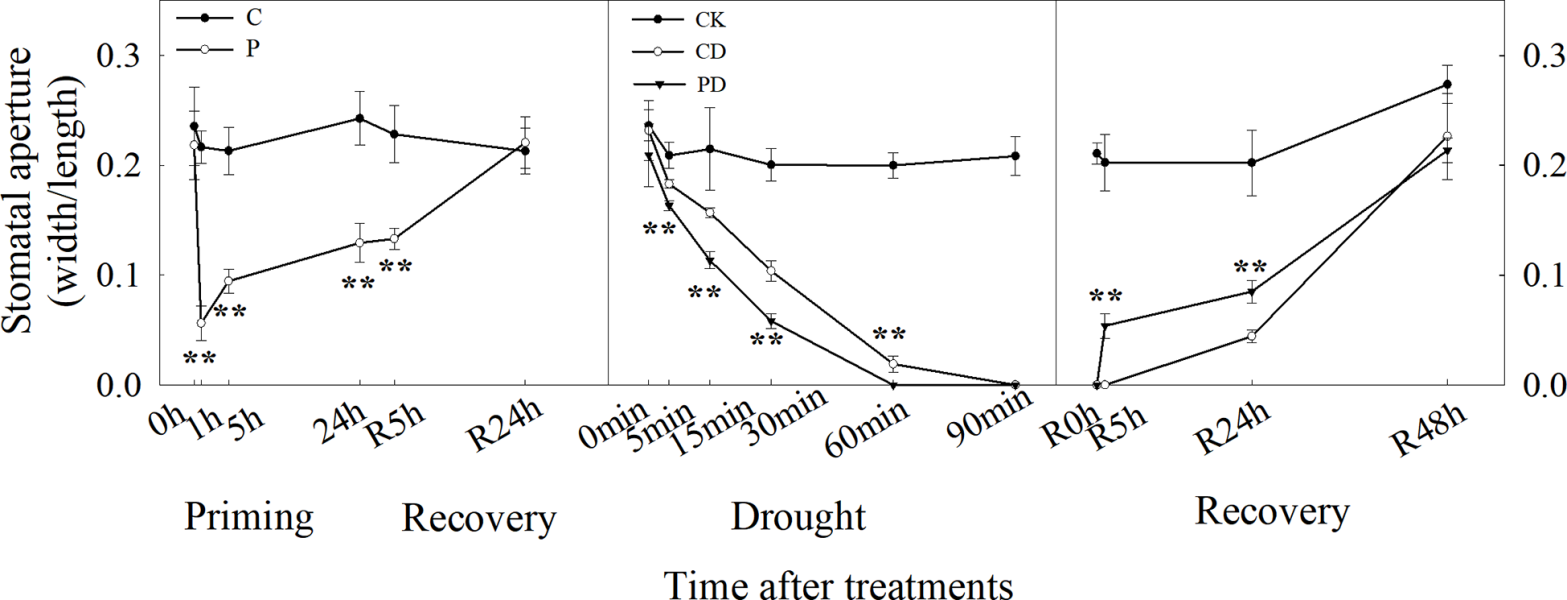
Dynamic responses of stomatal opening ratios during “drought priming-recovery-drought stress-recovery” in wheat. C, no drought priming; P, priming; CK, no drought priming + no drought stress; PD, priming + drought stress; CD, no priming + drought stress.*p < 0.05; **p < 0.01.

Time-course analysis showed that drought priming rapidly reduced SOR from 0 h to 1 h, then the stomatal opening rate gradually increased after 1 h under drought priming, and SOR showed no difference between P and C at 24 h after recovery from priming. Under drought stress, the SOR in CD and PD decreased, compared with CK, and decreased faster in PD than in CD. A faster recovery rate of stomatal opening was found in PD than in CD, which showed no significant difference after 48 h of recovery (**Figure 2**).

### 3.2 Guard cell ABA accumulation

**Figure 3:**
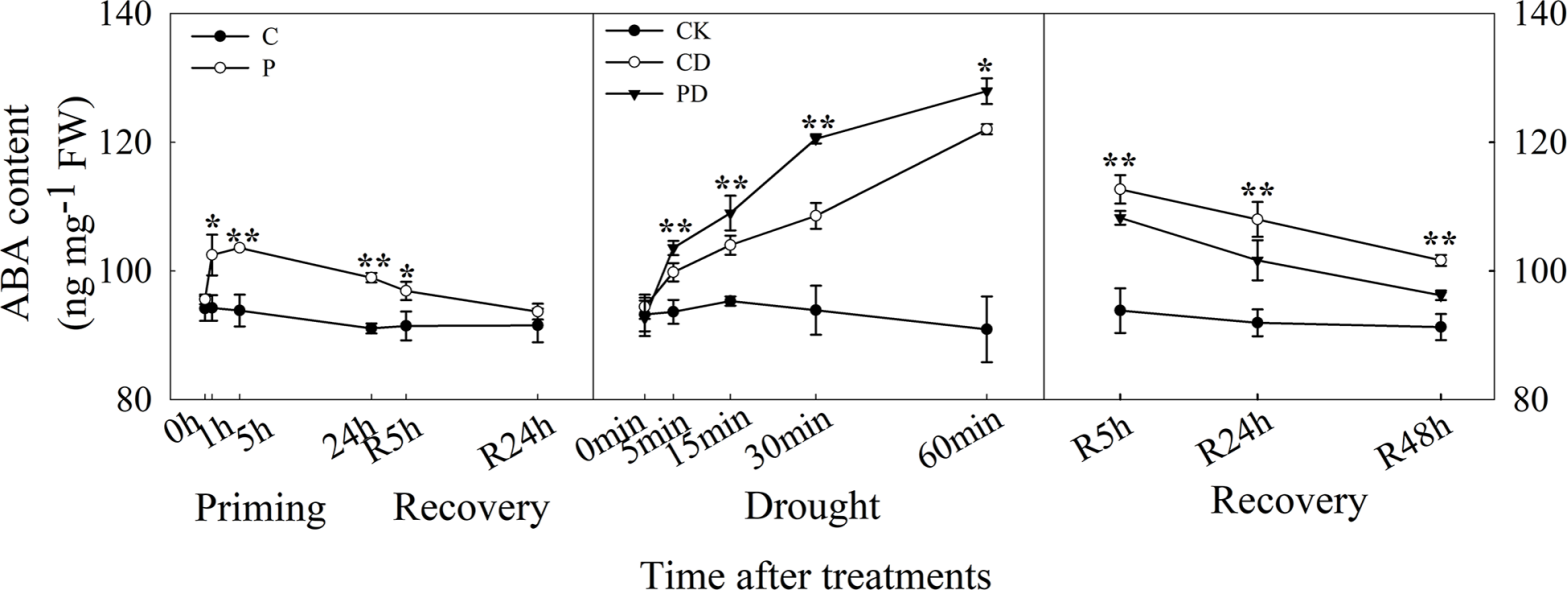
Dynamic changes in ABA content of guard cells during “drought priming-recovery-drought stress-recovery” in wheat. C, no drought priming; P, priming; CK, no drought priming + no drought stress; PD, priming + drought stress; CD, no priming + drought stress. *p < 0.05; **p < 0.01.

The ABA content increased rapidly within 1 h of drought priming and decreased after 5 h but was still higher than in the control. The ABA content significantly decreased after recovery, and no significant difference between P and C after 24 h recovery was found. Under drought stress, ABA content increased rapidly within 1 h, and was higher in PD than in CD. The ABA content decreased during the recovery, was higher in PD and CD than in CK, decreased much quicker in PD than in CD (**Figure 3**).

### 3.3 Guard cell calcium influx and signaling regulation

**Figure 4:**
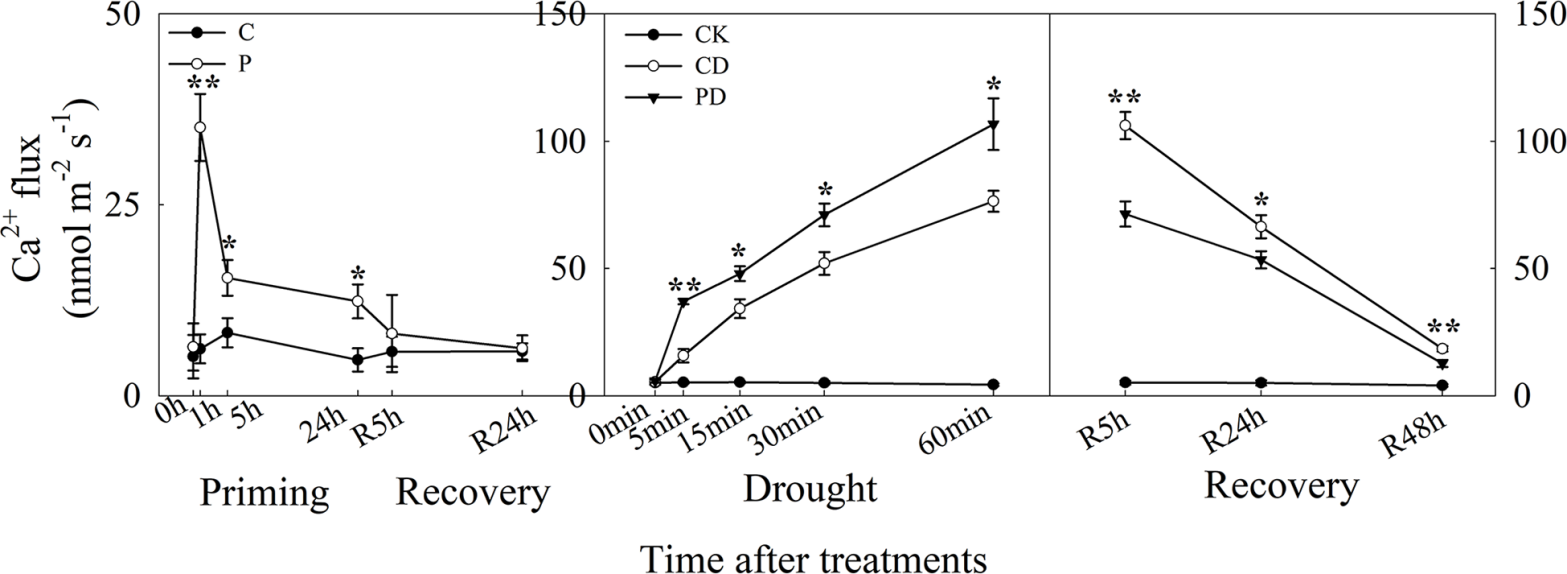
Dynamic responses of guard cells Ca^2+^ ion fluxes during “drought priming-recovery-drought stress-recovery” in wheat. C, no drought priming; P, priming; CK, no drought priming + no drought stress; PD, priming + drought stress; CD, no priming + drought stress. *p < 0.05; **p < 0.01.

The guard cells, Ca^2+^ influx was higher in P than in C, and ion flux reached a maximum at 1 h after priming. There was no difference between P and C during recovery. Under drought stress, Ca^2+^ influx increased significantly in both CD and PD, and higher in PD than CD. During recovery from stress, Ca^2+^ influx declined from R5 h to R48 h, and Ca^2+^ influx was significantly lower in PD than in CD (**Figure 4**).

**Figure 5:**
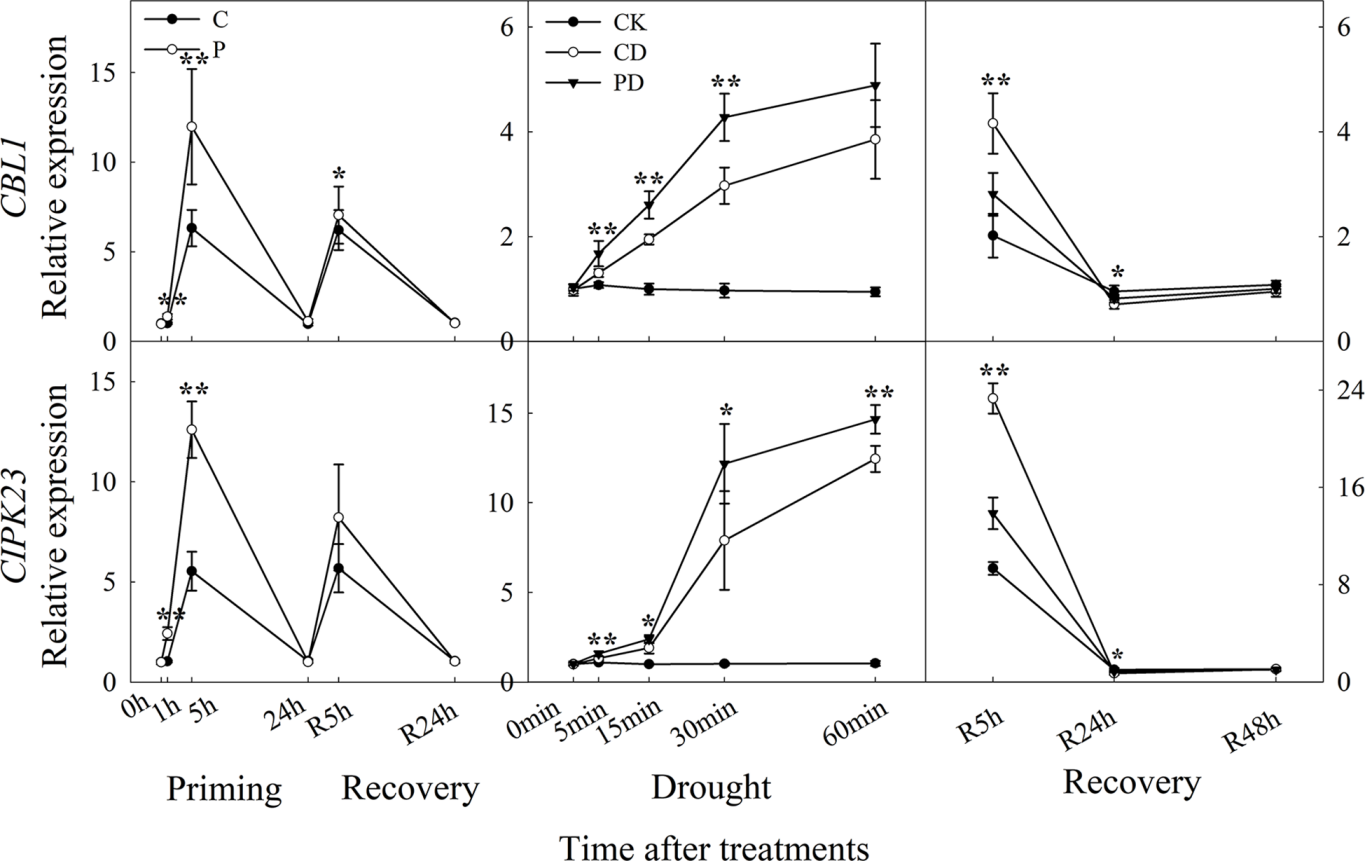
Dynamic responses of calcium ion signaling pathway related genes in leaf epidermis of wheat during “drought priming-recovery-drought stress-recovery”. C, no drought priming; P, priming; CK, no drought priming + no drought stress; PD, priming + drought stress; CD, no priming + drought stress. *p < 0.05; **p < 0.01.

The expression of *CBL1*, and *CIPK23* were higher expressed by drought priming. Only *CBL1* showed higher expression at R5h during recovery. During drought stress, *CBL1* and *CIPK23* were higher expressed in PD than in CD. Only *CBL1* and *CIPK23* showed lower expression in PD than in CD at R5 h and R24 h during recovery (**Figure 5**).

### 3.4 Guard cell ion flux and related channels

**Figure 6:**
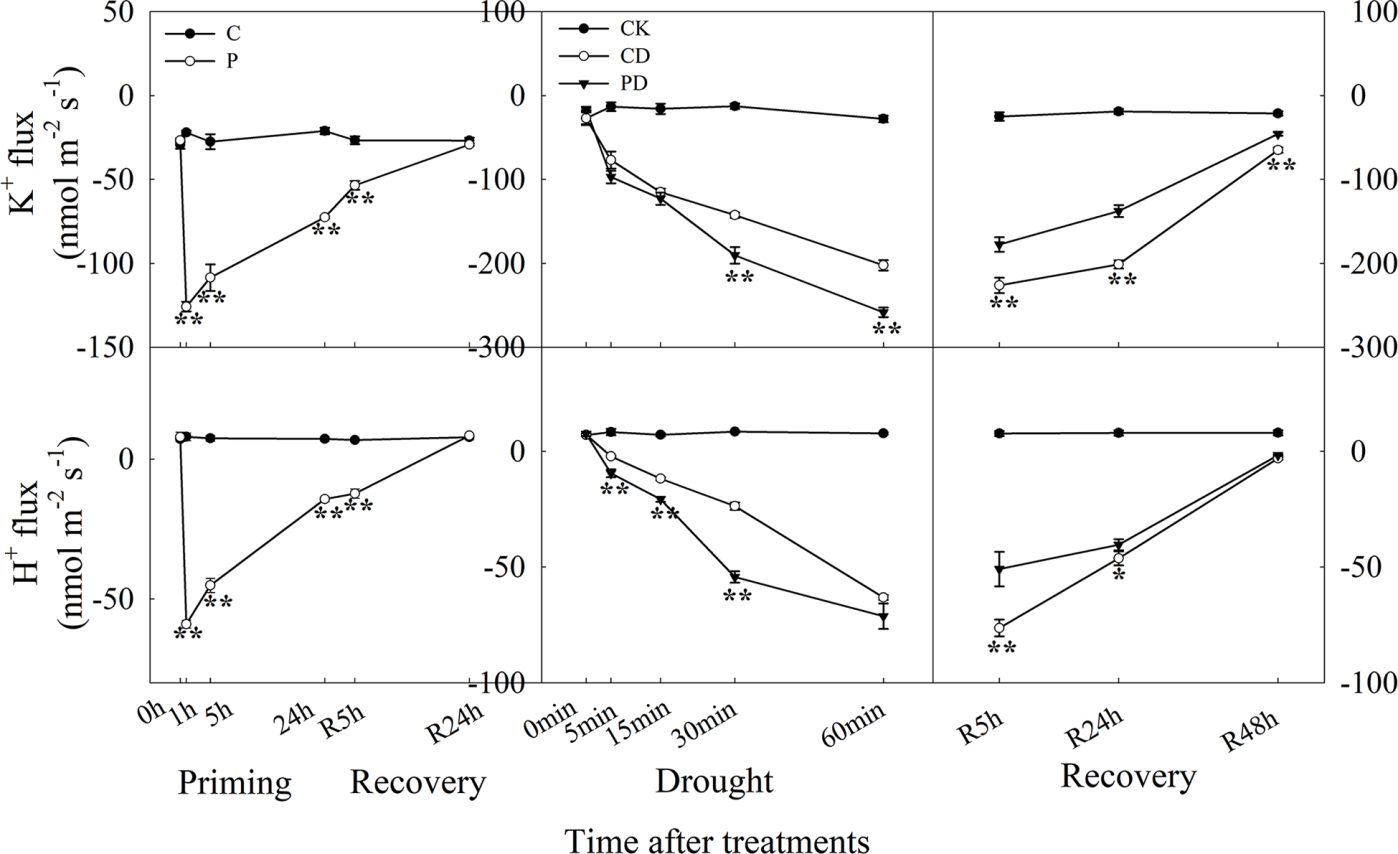
Dynamic responses of H^+^ and K^+^ fluxes in leaf epidermis of wheat during “drought priming-recovery-drought stress-recovery”. C, no drought priming; P, priming; CK, no drought priming + no drought stress; PD, priming + drought stress; CD, no priming + drought stress. *p < 0.05; **p < 0.01.

The efflux of K^+^ reached to the maximum within 1 h after drought priming, and efflux of K^+^ was higher in P than in C at R5 h during recovery. Drought stress promoted the rapid efflux of K^+^, and PD showed significantly higher efflux of K^+^ than the CD treatment within 1 h. During recovery, K^+^ efflux was significantly lower in PD than in CD. The H^+^ efflux showed a similar tendency as K^+^ during the priming-recovery-drought stress-recovery cycle (**Figure 6**).

**Figure 7:**
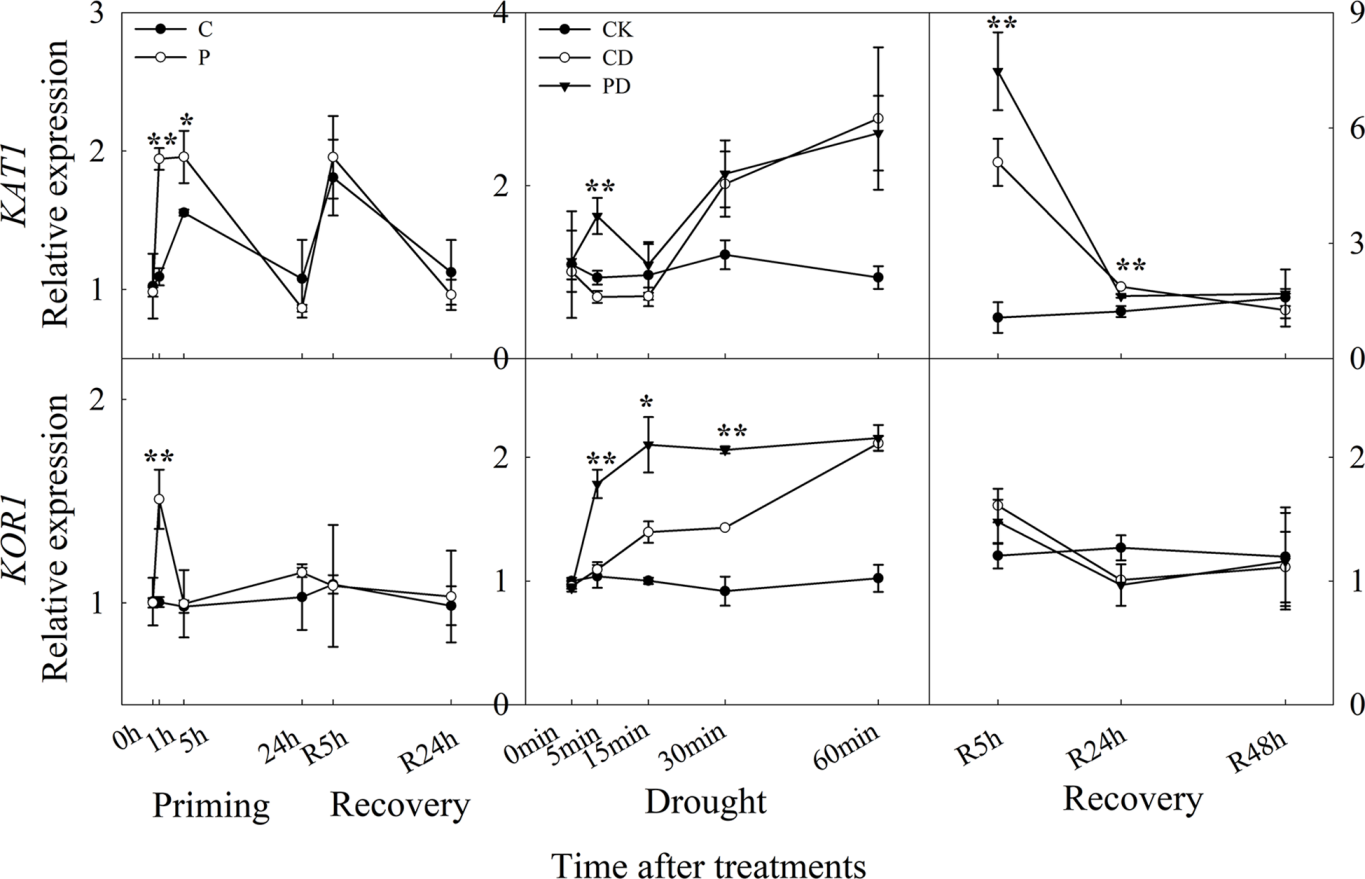
Dynamic responses of genes encoding K^+^ inward and outward channels in leaf epidermis of wheat during “drought priming-recovery-drought stress-recovery”. C, no drought priming; P, priming; CK, no drought priming + no drought stress; PD, priming + drought stress; CD, no priming + drought stress. *p < 0.05; **p < 0.01.

The expression of *KAT1* was significantly up-regulated, especially at 1 h and 5 h after drought priming, and the expression of *KOR1* was higher expressed at 1 h in P than in C. There was no significant difference between P and C during recovery. Under drought stress, the expression of *KAT1* was significantly higher expressed in PD than in CD at 5 min, while *KOR1* was higher expressed within 30 min. During recovery, only *KAT1* was found to be higher expressed in PD than in CD at R5 h and R24 h, the *KOR1* expression showed no significantly difference between PD and CD during recovery (**Figure 7**).

**Figure 8:**
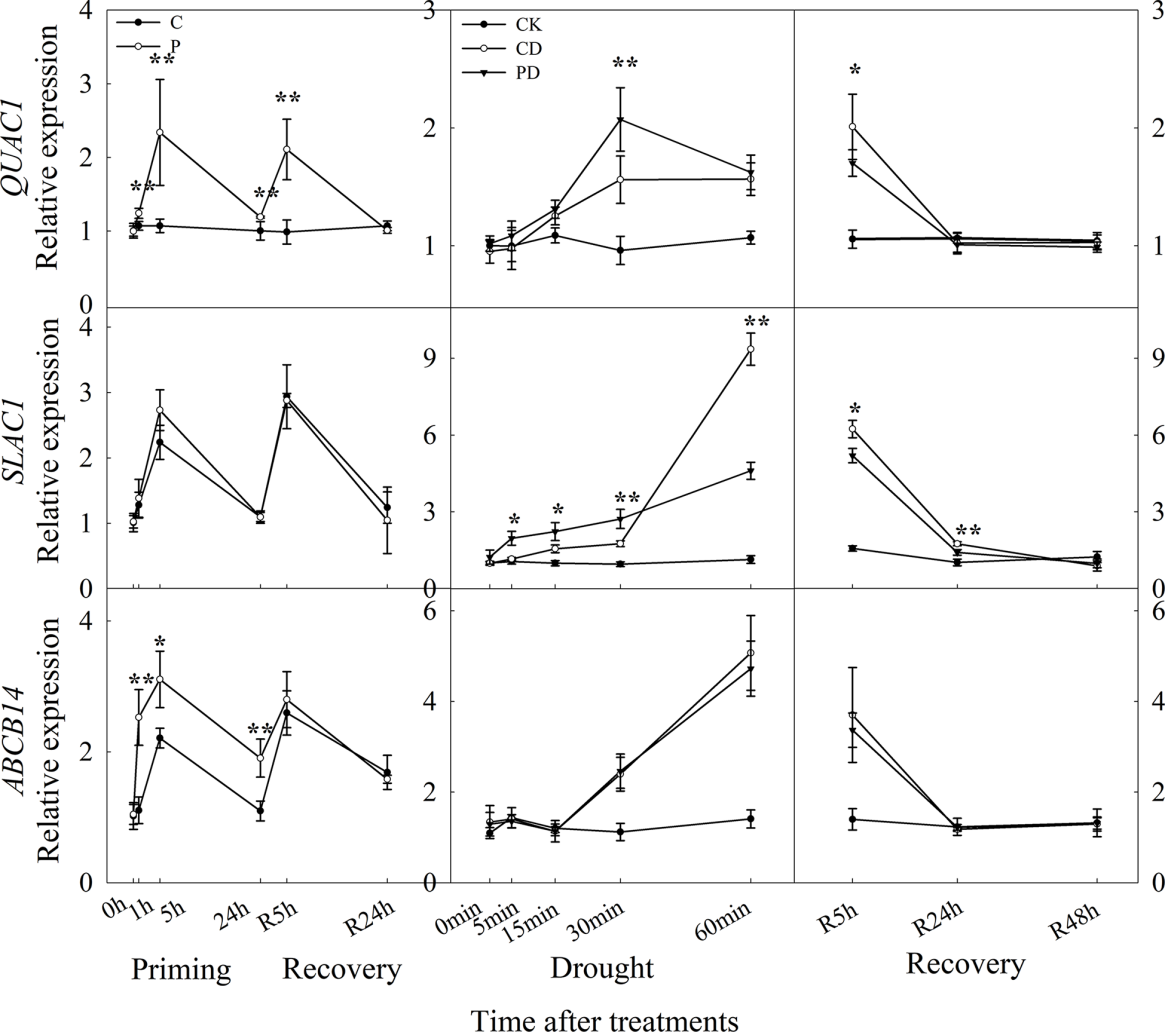
Dynamic responses of genes encoding anion fluxes channels in leaf epidermis of wheat during “drought priming-recovery-drought stress-recovery”. C, no drought priming; P, priming; CK, no drought priming + no drought stress; PD, priming + drought stress; CD, no priming + drought stress. *p < 0.05; **p < 0.01.

**Figure 9:**
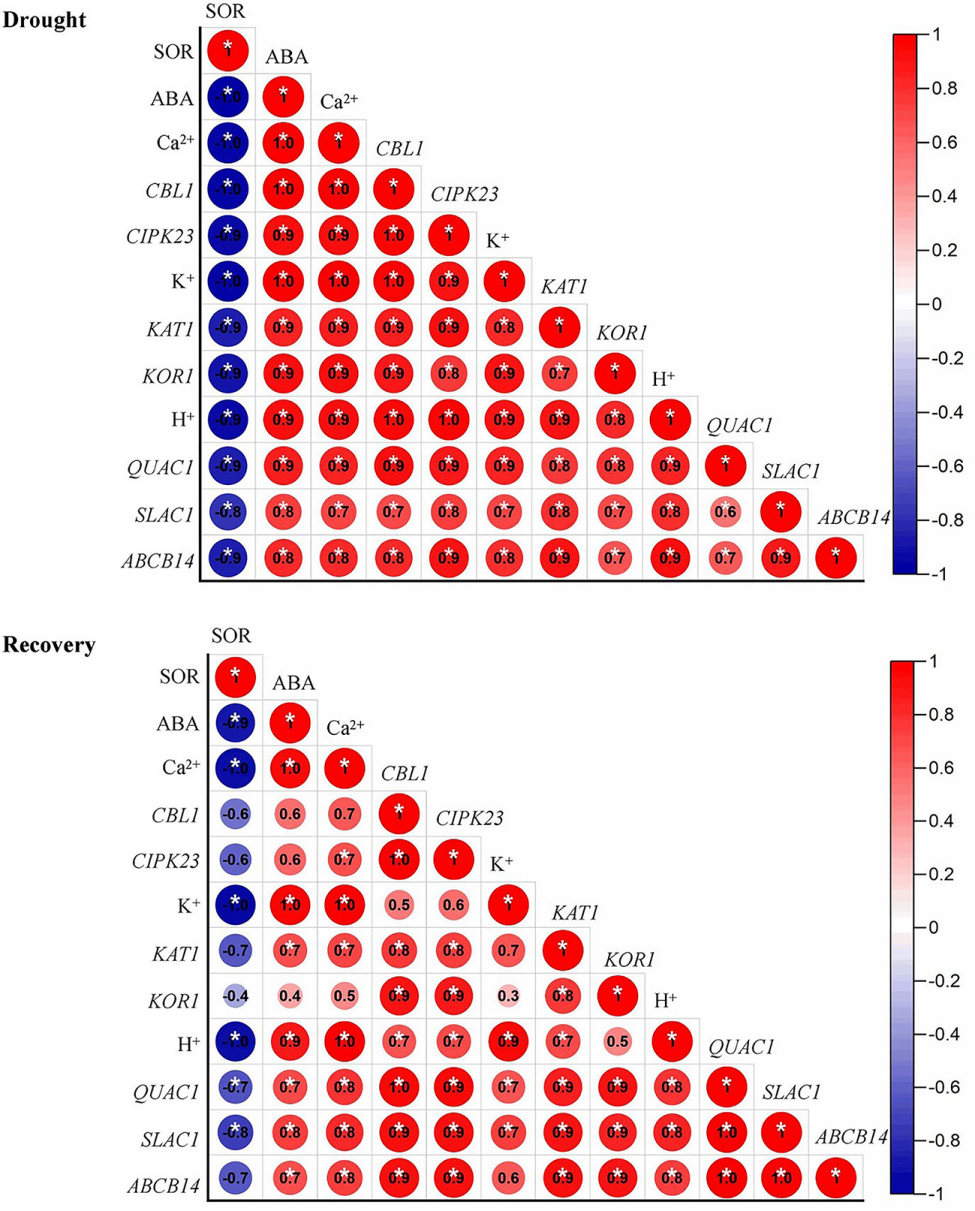
Correlation analysis among physiological parameters under drought stress **(A)** and recovery **(B)**. SOR, stomatal opening rate. Correlations are expressed using Pearsons coefficient.

The expression level of *QUAC1, ABCB14* were significantly induced by drought priming, but only *QUAC1* was higher expressed in P than in C during recovery. Under drought stress, *SLAC1* was higher expressed in PD than in CD within 60 min, and *QUAC1* was higher expressed in PD than in CD at 30 min under drought stress. Under recovery, only *SLAC1* was lower expressed in PD than in CD at R5 h and R24 h. There were no significant differences in *ABCB 14* between PD and CD during drought stress and recovery (**Figure 8**). The correlation analysis found that, the reduced stomatal opening rate showed negative with ABA content, Ca^2+^ flux, K^+^ flux, H^+^ flux, expression of *CBL1*, *CIPK23*, *KOR1* under drought stress, The stomatal reopening rate showed negative with ABA content, Ca^2+^ flux, K^+^ flux, H^+^ flux, and the expression of *QUAC1* and *SLAC1* under post-drought recovery (**Figure 9**).

## 4 Discussion

Stomata play an important role in regulating plant water use-efficiency and improving drought tolerance (Polle et al., 2019; Shohat et al., 2021). Previous studies found that drought priming enhanced drought tolerance by improving both photosynthesis and biomass accumulation (Wang et al., 2014a; Wang et al., 2021). Here, consistent with earlier studies, drought primed plants showed higher drought tolerance by enhancing photosynthesis and biomass accumulation (**Table 1**). However, the dynamic stomatal behavior response to a priming-recovery-drought stress-recovery cycle has not been investigated. To investigate the role of stomatal regulation during this cycle, the *in-situ* stomatal movement characteristics were monitored, and potential mechanisms were revealed.

### 4.1 Stomatal of primed wheat plants response faster to drought stress and recovery

It has been found that dehydration stressed *Arabidopsis* plants partially closed stomatal apertures during the recovery period, which contributed to reduced transpiration during a reoccurring dehydration stress event (Virlouvet and Fromm, 2015). Here, dynamic monitoring of stomatal behavior found that the SOR decreased by drought priming during the first 2 hours and remained lower than the control in the first 2 hours under priming-after recovery. This was consistent with another study in tomato which found that there may exist an “after-effect” of drought during recovery, as the stomata could not fully recover (Visentin et al., 2020). Under the reoccurring drought stress treatment, stomatal movement in primed plants closed much quicker than in non-primed plants. However, primed plants showed an earlier reopening of stomata than did non-primed plants in the post- drought recovery. The faster response of stomata to post-drought recovery may related to the stress memory effect. It is suggested from this study that the faster stomatal response of primed plants to the reoccurred drought stress and post-drought recovery periods lead to better CO_2_ assimilation and water-use efficiency, which contributed to enhance drought tolerance.

### 4.2 ABA and Ca^2+^ are involved in priming-induced stomatal movement during drought stress and post-drought recovery

ABA is an important signal molecule for plant stomatal regulation (Yao et al., 2021). Our previous study found that ABA mediated drought priming induced drought tolerance in wheat (Wang et al., 2021). Here, ABA content significantly increased in primed plants and decreased during the recovery stage compared with control. This was consistent with the decreased stomatal SOR after drought priming and still lower SOR during early priming after recovery, compared with control. ABA increased significantly within 1 h during drought stress, with higher content in primed plants, and ABA content was much lower in primed plants during post-drought recovery, compared with non-primed plants. The ABA content was consistent with the stomatal behavior characteristics during the drought priming-recovery-drought stress-recovery cycle.

The ABA signaling pathway regulates the increase of intracellular Ca^2+^, which further targets the activation and inactivation of protein kinase/phosphatases in ion channels, as well as the regulation ion flow in guard cell leading to stomatal closure (Qi et al., 2018). Here, the Ca^2+^ influx was much higher in primed plants under priming and drought stress but much lower in non-primed plants during post-drought recovery. The Ca^2+^ influx showed similar tendency as the ABA content under the different treatments, suggesting that Ca^2+^ influx channels were activated to regulate Ca^2+^ influx during the drought priming-recovery-drought stress-recovery cycle.

Calcineurin B-like protein (CBL) has been shown to interact with CBL-interacting protein kinase (CIPK) which play important roles in the Ca^2+^ signaling pathway (Sanyal et al., 2020). *CIPK23* regulates light induced stomatal regulation in *Arabidopsis* (Inoue et al., 2020). It has been demonstrated that the *CBL1-CIPK23* is involved in ABA-mediated drought tolerance in wheat (Cui et al., 2018). In this study, the expressions of *CBL1* and *CIPK23* in guard cells were significantly up-regulated by drought priming and were expressed higher in primed plants than non-primed plants under drought stress. They were expressed significantly lower in primed plants than in non-primed plants under post-drought recovery. The results indicated that the Ca^2+^ signaling pathway in primed plants was more activated under drought stress and less activated under recovery, which correlated with the stomatal regulation pattern.

### 4.3 Ion channels are involved in drought priming induced stomatal movement

Stomatal closure is dependent on the activation of ion channels in the vacuolar and plasma membranes. Guard cells K^+^ ions and anions flow through potassium- and anion channels, resulting in water efflux, reduced turgor pressure, and ultimately led to stomatal closure (Kim et al., 2010; Wang et al., 2014b). In this study, primed plants showed higher efflux of K^+^ and H^+^ under drought stress, and lower efflux of K^+^ and H^+^ under recovery, compared with non-primed plants. It is known that guard cell inward-rectifying K^+^ channels (KAT) and K^+^ outward-rectifying channels (KOR) mediate K^+^ flux in guard cells (Hwang et al., 2013). The expression of inward K^+^ channels was found to be reduced by ABA (Roberts and Snowman, 2000). It has been found that the endogenous ABA content showed positive correlation with the activating the outward K^+^ channel, promoting K^+^ efflux across guard cells, causing loss of cell turgor and of stomatal closure (Roelfsema et al., 2012).

Here, both *KAT1* and *KOR1* were upregulated by drought priming, which lead to increased efflux of K^+^. The efflux of H^+^ hyperpolarizes the membrane and leads to K^+^ uptake *via* activation of inward K^+^ rectifying channels (Daszkowska-Golec and Szarejko, 2013). Here, the activating of K^+^ release channels *KOR1* were higher expressed in primed plants which lead to the higher efflux of K^+^ than in non-primed plants under drought stress. However, the expression of *KAT1* was much higher in primed plants under recovery, and then lead to lower K^+^ efflux.

Previous studies have shown that anion efflux of guard cells involves anion channels, including the slow activating anion channel (SLAC1) and fast activating anion channel (QUAC1) (Hedrich et al., 1990). The coordinated activation of both *SLAC1* and *QUAC1* anion channels are required for stomatal closure in *Arabidopsis* (Jalakas et al., 2021). Over-expression of *SLAC1* lead to stomatal closure (Zhang et al., 2016), and mutations in the *SLAC1* mutant slowed stomatal opening (Laanemets et al., 2013). Here, only *QUAC1* were highly induced by drought priming. The primed plants showed higher expression of *SLAC1* under drought stress, and lower expression of *SLAC1* under recovery, compared to non-primed plants. *ABCB14* which encodes the malate ion channel was induced by drought priming only, while no differences between primed plants and non-primed plants under drought stress and recovery were found.

## 5 Conclusion

**Figure 10:**
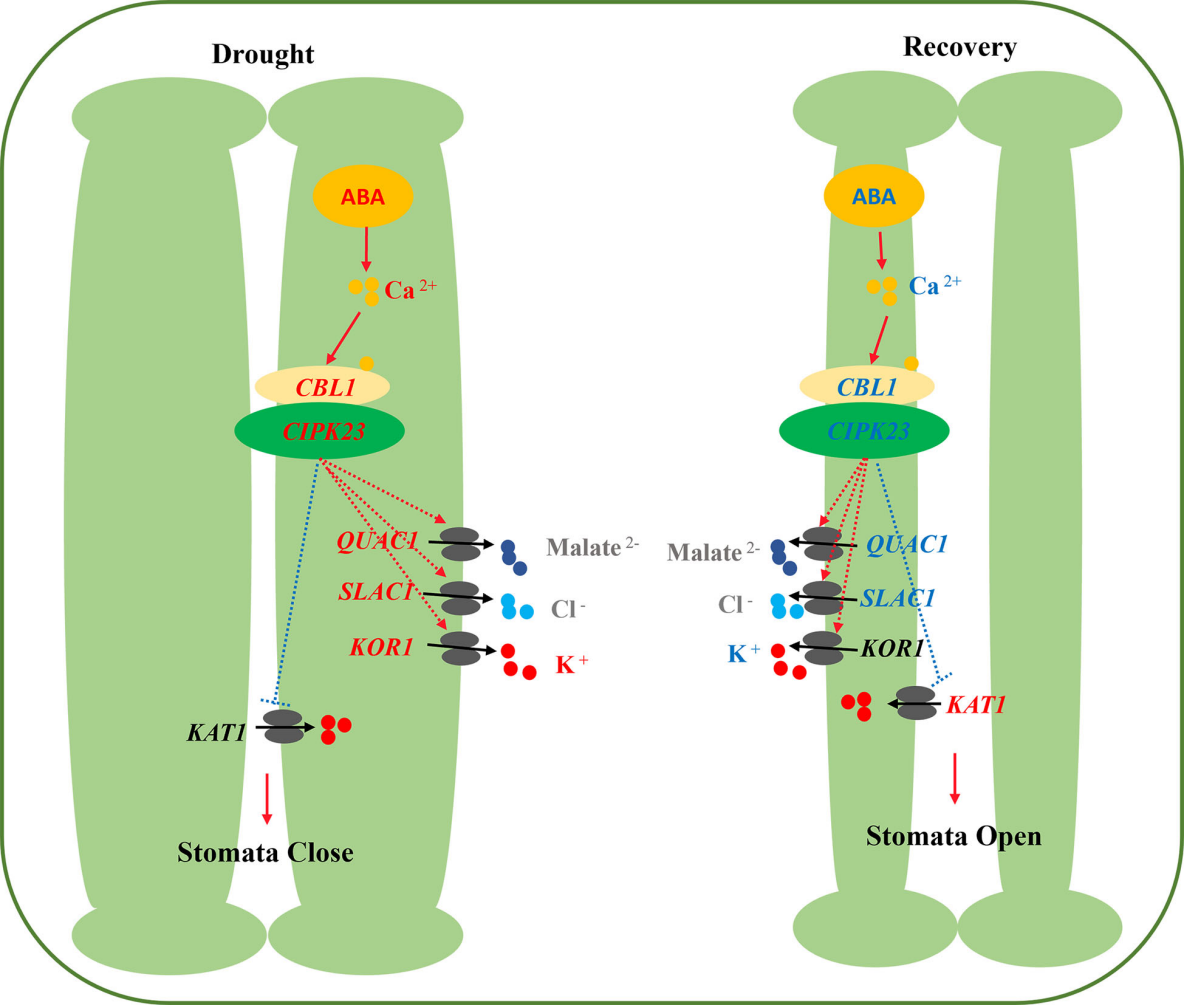
Physiological mechanisms of stomatal movement induced by drought priming. Red indicate the higher expression and blue indicate the lower expression in PD than in CD.

The stomata of primed plants responded much faster to reoccurring drought stress and recovery (i.e., they closed faster with drought stress and reopened faster with post-drought recovery). Compared with non-primed plants, drought priming induced higher accumulation of ABA in guard cells, activating the Ca^2+^ influx and the Ca^2+^ signaling pathway, as well as the activation of S-type and R-type anion channels (*SLAC1* and *QUAC1*) that lead to the efflux of anions, and the activation of the guard cell K^+^ efflux channel *KOR1* lead to enhanced K^+^ efflux, contributing to accelerated stomatal closure in primed plants under drought stress. Under recovery from drought stress, primed plants showed lower ABA contents, lower Ca^2+^ influx and a lower Ca^2+^ signal transduction in guard cells. Lower expression of *SLAC1* and higher expression of the K^+^ influx channels *KAT1* lead to less K^+^ efflux and promoted reopening of stomata (**Figure 10**). The study will deeply illustrate the underlying mechanisms of stomatal regulation in drought priming induced drought tolerance of wheat, and help breeding new drought resistant wheat cultivar.

## Data availability statement

The raw data supporting the conclusions of this article will be made available by the authors, without undue reservation.

## Author contributions

XW designed the experiment, supervised the whole project, and wrote the manuscript. MY and XW performed the experiments and biochemistry analyses, and analyzed the data; JH, ZS, QL, JC, QZ involved in the experiment setup and biochemistry analyses; BW, DJ and XW assisted with the discussion and provided critical feedback on the article. All authors contributed to the article and approved the submitted version.
