# Supplementary material for: Drought priming mechanisms in wheat elucidated by in-situ determination of dynamic stomatal behavior
Source: Front Plant Sci. 2023 Feb 17;14:1138494. doi: 10.3389/fpls.2023.1138494 (PMC9983753; doi:10.3389/fpls.2023.1138494)
Supplement: Supplementary file 1 [file Table_1.docx]

Supplementary Material

**Drought priming mechanisms in wheat elucidated by in-situ determination of dynamic stomatal behavior**

Xiao Wang*, Mengxiang Yang, Jiawei He, Qing Li, Jian Cai, Qin Zhou, Bernd Wollenweber, Dong Jiang*

* **Correspondence：**

Correspondence author: Dong Jiang Tel and Fax: +86 25 84399627

E-mail: [jiangd@njau.edu.cn](mailto:jiangd@njau.edu.cn)

Xiao Wang Tel and Fax: +86 25 84399627

E-mail: [xiaowang@njau.edu.cn](mailto:xiaowang@njau.edu.cn)

**Supplementary Tables**

**Supplementary Table S1.** Primers used for real time quantitative PCR

| Gene | Forward primer 5’-3’ | Reverse primer 5’-3’ |
| --- | --- | --- |
| *Actin* | GACGCACAACAGGTATCGTGTTG | AGCGAGGTCAAGACGAAGGATG |
| *KAT1* | TTCTGGGATGGGCTTCACAT | CKAGAATCTGTAGCGGGGAT |
| *KOR1* | CAAGGCAGTGCAGTTGATCA | TCTCKTCTTGACKGTCTTCG |
| *ACA5* | GTCACATTAACGCTCGCKTT | CKATTGTCTCGCATGCAGAA |
| *SLAC1* | GACGACGGGTGGTTCTACAC | GTAGAGCAGCGACTCKACKT |
| *QUAC1* | CAACKACGGCTATTTCTCGG | TCTTGAATGAGGGCAGAGCA |
| *ABCB14* | TGCAGACTCGGAGAACATTG | TGTTGTAACACKGCGATCAT |
| *NCED1* | CKCAGCACTAATCGATTCK | CKGCTAACTGTATCKATGC |
| *NCED2* | GGAGATGGAAAGAGGAAGTCG | GAAGCAAGTGTGAGCTAAC |
| *CML16* | CAGCAACAAACCCAAGAAGGA | CCAACAGGGACTGACAACTAA |
| *CBL1* | ACGTCTACATTGTGCTCGGA | AGGTGAAAGGAGGAGAAGCK |
| *CIPK23* | TCAAGCACAAGATGATCGCG | TATCTTTGTCKTGCTGGCKA |
